# The association between weight-adjusted-waist index and sarcopenia in adults: a population-based study

**DOI:** 10.1038/s41598-024-61928-0

**Published:** 2024-05-13

**Authors:** Haojing Zhou, Hai Su, Yichen Gong, Lei Chen, Lihan Xu, Guoqian Chen, Peijian Tong

**Affiliations:** 1 Zhejiang Chinese Medical University, Hangzhou, Zhejiang Province China; 2 College of Stomatology, Hangzhou Normal University, Hangzhou, Zhejiang Province China; 3 The First Affiliated Hospital of Zhejiang Chinese Medical University (Zhejiang Provincial Hospital of Chinese Medicine), Hangzhou, Zhejiang Province China

**Keywords:** Cross-sectional studies, NHANES, Obesity, Sarcopenia, Weight-adjusted-waist index, Obesity, Geriatrics, Public health

## Abstract

This study aims to investigate the relationship between weight-adjusted-waist index (WWI), a new body index, and sarcopenia, while also assessing the potential of WWI as a tool for screening sarcopenic patients. The cross-sectional study involved adults who possessed complete data on WWI and appendicular skeletal muscle mass from the 1999–2006 and 2011–2018 National Health and Nutrition Examination Surveys. Weighted multivariate regression and logistic regression analyses were employed to explore the independent relationship between WWI and sarcopenia. The study included 26,782 participants. The results showed that WWI demonstrated a positive correlation with sarcopenia risk. In the fully adjusted model, with each 1 unit increase in WWI, the risk of developing sarcopenia rose 14.55 times higher among males (OR: 14.55, 95% CI 12.33, 17.15) and 2.86 times higher among females (OR: 2.86, 95% CI 2.59, 3.15). The optimal cutoff values of WWI for sarcopenia were 11.26 cm/√kg for males and 11.39 cm/√kg for females. Individuals with a higher WWI have an increased risk of developing sarcopenia, and a high WWI functions as a risk factor for sarcopenia. Assessing WWI could assist in identifying individuals at risk of sarcopenia.

## Introduction

Sarcopenia refers to the gradual and progressive decline in skeletal muscle mass and strength, frequently accompanied by a decrease in physical function^1,2^. This condition is commonly recognized as a significant syndrome associated with aging. Greater awareness of sarcopenia suggests that the decrease in muscle strength and mass may commence early in life^3^. Sarcopenia independently predicts several clinically significant adverse outcomes, including a higher risk of fractures, reduced quality of life, impaired mobility, and increased mortality rates^4–6^. The presence of sarcopenia escalates the risk of hospitalization and amplifies the cost of care associated with hospital stays^7^. The prevention and treatment of sarcopenia are gradually receiving increased attention. The SARC-F scale is presently a common tool for screening sarcopenia patients; however, it suffers from low sensitivity, making it prone to missing suspicious cases^8^. Furthermore, the SARC-F scale is relatively intricate, and there exists a dearth of simple indicators for assessing sarcopenia.

The weight-adjusted-waist index (WWI) is a newly introduced anthropometric measure obtained by standardizing waist circumference (WC) to body weight^9^. Similar to body mass index (BMI), a higher WWI score indicates elevated levels of obesity. Throughout the progression of sarcopenia, there is a tendency for fat to increase, either relatively or absolutely. The clinical and functional disease characterized by the coexistence of obesity, excess fat, and sarcopenia is termed sarcopenic obesity^10^. Prior studies have established an independent association between WWI and sarcopenic obesity in specific cohorts, such as patients with type 2 diabetes mellitus and males undergoing maintenance hemodialysis^11,12^. Notably, WWI displays a stronger correlation with sarcopenic obesity in elderly males compared to other anthropometric indices, including waist-to-height ratio, BMI, and WC^13^. Furthermore, among middle-aged and elderly individuals, there exists a correlation between muscle mass, a key indicator for diagnosing sarcopenia, and WWI^14–16^. However, the correlation between WWI and sarcopenia remains unexplored.

This study aimed to achieve the following objectives: firstly, to evaluate the incidence of sarcopenia and its incidence in different age groups. Secondly, to investigate the relationship between WWI and sarcopenia. Lastly, to determine the critical cutoff value of the WWI index for the evaluation of sarcopenia.

## Methods

### Data source and study population

The data were obtained from the National Health and Nutrition Examination Survey (NHANES), a nationally conducted cross-sectional survey conducted by the National Center for Health Statistics. NHANES aims to collect information on potential health risk factors and the nutritional status of non-institutionalized civilians in the United States. A complex stratified multistage probability cluster sampling design was utilized to acquire a representative sample of the entire United States population^17^. The NHANES study protocols received approval from the Research Ethics Review Board of the NCHS. All participants gave their written informed consent. Comprehensive details about the NHANES study design and data are publicly available at https://www.cdc.gov/nchs/nhanes/.

**Figure 1:**
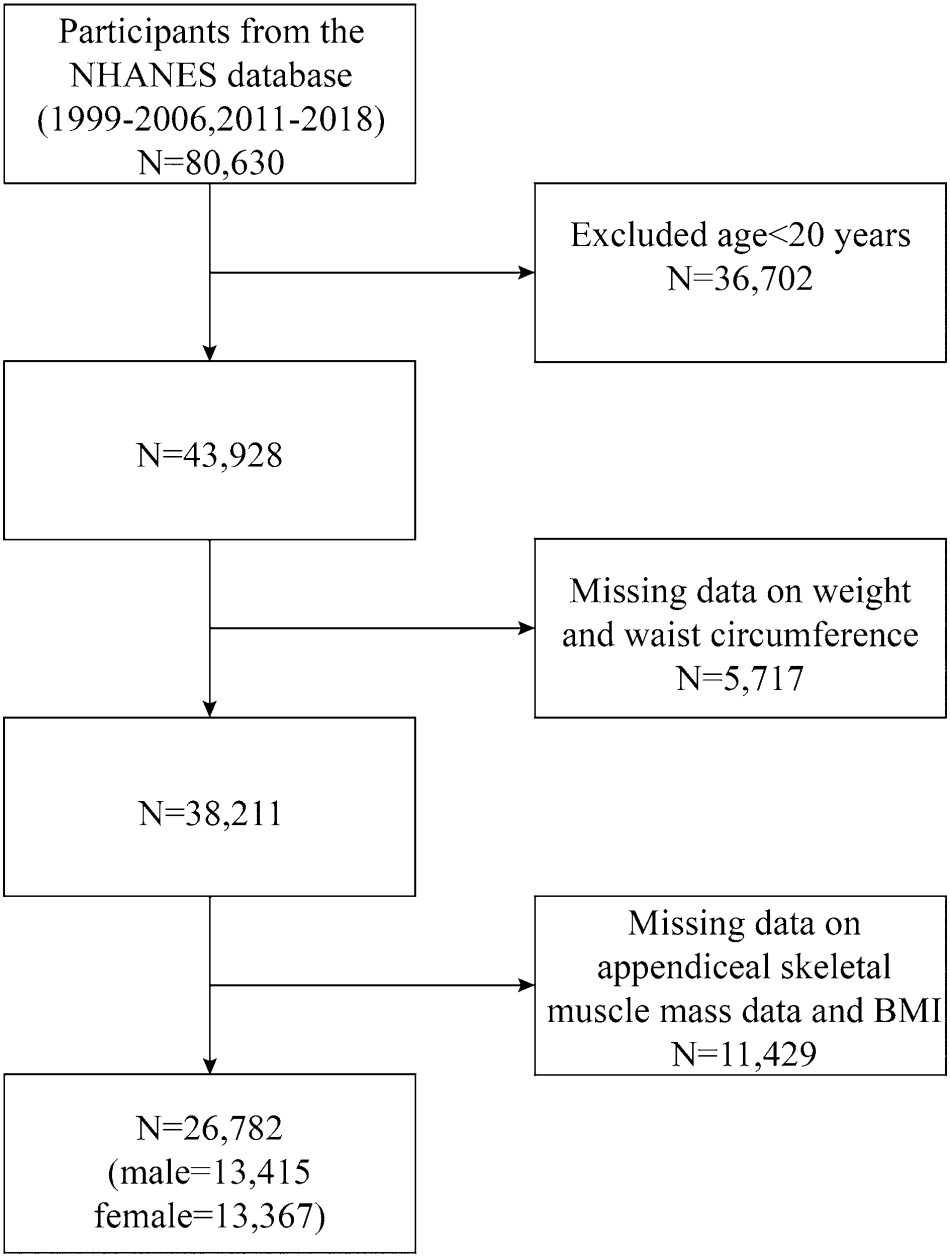
Flowchart of population included in our final analysis.

Our study utilized data from NHANES survey cycles from 1999–2006 and 2011–2018, as these cycles were the only ones containing information on appendicular skeletal muscle mass (ASM) and WWI. Given that this study investigates the correlation between WWI and sarcopenia in adults, we adhered to the methodology of similar studies^18,19^ by excluding participants under the age of 20 and those lacking WWI and ASM data. Initially, 80,630 participants were enrolled. However, following the exclusion of individuals under 20 years of age (n = 36,702), those with missing WWI data (n = 5,717), and missing ASM data and BMI (n = 11,429), our final analysis comprised 26,782 participants. The cohort comprised 13,415 male and 13,367 female (Fig. 1).

### Assessment of WWI

WWI, derived from WC and weight, functions as an estimator for obesity. Skilled health technicians collected body measurement data concerning WC and weight at the mobile examination center. WWI (cm/√kg) was calculated as $\frac{WC}{\sqrt{Weight}}$, rounded to two decimal places. Within our analysis, we treated WWI as a continuous variable and subsequently grouped participants based on WWI quartiles for further examination. WWI was employed as an exposure variable in our study.

### Definition of sarcopenia

According to the Foundation for National Institutes of Health Osteoarthritis Biomarkers study recommendations, individuals are considered to have sarcopenia if their sarcopenia index falls below 0.789 for men and 0.512 for women. Many similar studies have used this criterion to define sarcopenia^18–20^. The sarcopenia index is calculated as the ratio of total ASM (kg) to BMI (kg/m^2^)^21^. ASM included all non-fat and non-bone tissues, while ASM specifically comprised the combined lean soft tissues of the extremities. NHANES utilized dual-energy X-ray absorptiometry (DEXA) for the measurement of ASM.

### Covariates

Our study incorporated covariates that might influence the relationship between WWI and sarcopenia. These included gender (male/female), age (year), race (non-Hispanic White/non-Hispanic Black/Mexican American/other races), education level (less than high school/high school/more than high school), BMI (kg/m^2^), intake of energy (kcal/day), intake of protein (gm/day), albumin (mg/L), creatinine (umol/L), total cholesterol (mmol/L), high density lipoprotein cholesterol (mmol/L), low density lipoprotein cholesterol (mmol/L), triglyceride (mmol/L), smoking status (yes/no), alcohol status (yes/no), hypertension (yes/no), and diabetes (yes/no).

Energy and protein intake were calculated by averaging the intake across day 1 and day 2. Smoking status was determined based on whether one had smoked at least 100 cigarettes in life. Participants who had at least 12 alcohol drinks per year were considered drinkers. Hypertension diagnosis was established through patient self-report and blood pressure measurements (systolic blood pressure ≥ 140 mm Hg or diastolic blood pressure ≥ 90 mm Hg)^22^. Diabetes was identified through self-report and glycohemoglobin levels (≥ 6.5%)^23^. In subgroup analysis, BMI was grouped as < 18.5, 18.5–24.9, 25–29.9, and 30–34.9, 35–39.9, ≥ 40 kg/m^2^, corresponding to underweight, normal weight, overweight, obesity class I, obesity class II, and obesity class III, respectively.

### Statistical analysis

There are notable disparities in the diagnostic criteria for sarcopenia between males and females. Consequently, this study is divided into groups based on gender to examine the relationship between WWI and sarcopenia. Continuous variables were presented as mean ± standard deviation, whereas categorical variables were represented as percentages. Differences between groups were evaluated using a weighted Student’s t-test (for continuous variables) or a weighted chi-square test (for categorical variables). Logistic regression was employed to examine the association between WWI and sarcopenia, utilizing the corrected odds ratio (OR) and the corresponding 95% confidence intervals (CI) to delineate the relationships. In model 1, no covariates were adjusted. In model 2, age, race, and education level were adjusted. Model 3 was adjusted for age, race, education level, BMI, intake of energy, intake of protein, albumin, creatinine, total cholesterol, high density lipoprotein cholesterol, low density lipoprotein cholesterol, triglyceride, smoking status, alcohol status, hypertension and diabetes. Subgroup analyses were conducted to investigate the relationship between WWI and sarcopenia, stratified by gender (male/female), age groups (20–39/40–59/ ≥ 60 years), BMI categories (underweight/normal weight/overweight/obesity class I/obesity class II/obesity class III), hypertension (yes/no), and diabetes (yes/no), considering these factors as predetermined potential effect modifiers. An interaction term was introduced to assess the variations in associations among subgroups. The predictive capacity of WWI for sarcopenia was assessed using Receiver Operating Characteristic (ROC) curve analysis, obtaining area under the curve (AUC), sensitivity, and specificity values. In general, an AUC value of 0.5 indicates a lack of discrimination, while a range of 0.7–0.8 is deemed acceptable, 0.8–0.9 is regarded as excellent, and values exceeding 0.9 are considered outstanding^24^. Missing values for continuous variables were imputed with the mean, and categorical variables were imputed with the mode, restricted to available cases. Statistical analyses were conducted using R version 3.4.3 (http://www.R-project.org, The R Foundation) and Empower software (www.empowerstats.com; X&Y solutions, Inc., Boston, MA). Statistical significance was defined as a two-sided *P* value < 0.05.

### Ethical approval

These studies involving humans have been approved by the Ethics Review Board of the National Center for Health Statistics. The studies were conducted in accordance with local legislation and institutional requirements. According to national legislation and institutional requirements, participants or their legal guardians/next of kin do not require written informed consent. Detailed information is available at https://www.cdc.gov/nchs/nhanes/irba98.htm.

## Results

### Baseline characteristics of participants

Tables 1 and 2 display the inclusion of 26,782 participants in the study, comprising 13,415 males and 13,367 females. Among male participants, the mean WWI measured 10.73 ± 0.78, with a sarcopenia incidence of 12.29% (6.17% in the 20–39 age group, 10.32% in the 40–59 age group, 30.11% in the ≥ 60 age group). The average WWI was 11.69 ± 0.54 in patients with sarcopenia and 10.60 ± 0.71 in patients without sarcopenia. Among female participants, the mean WWI was recorded as 11.03 ± 0.85, with a sarcopenia incidence of 11.33% (5.30% in the 20–39 age group, 11.09% in the 40–59 age group, 24.23% in the ≥ 60 age group). The average WWI was 11.87 ± 0.74 in patients with sarcopenia and 10.92 ± 0.80 in patients without sarcopenia. Statistically significant differences existed between the sarcopenic and non-sarcopenic populations across most covariates, irrespective of gender.

**Table 1:** Baseline characteristics of male study participants.

**Characteristics**	Overall	Non-sarcopenia	Sarcopenia	*P* value
N	13415	11766	1649(12.29%)	< 0.001
20–39 years	5675	5325	350(6.17%)	< 0.001
40–59 years	5213	4675	538(10.32%)	< 0.001
≥ 60 years	2527	1766	761(30.11%)	< 0.001
WWI (cm/√kg)	10.73 ± 0.78	10.60 ± 0.71	11.69 ± 0.54	< 0.001
Age (years)	44.66 ± 16.41	43.06 ± 15.55	56.06 ± 17.79	< 0.001
Race/ethnicity, N (%)				< 0.001
Non-Hispanic White	5873(43.66%)	5181 (44.03%)	676 (40.99%)	
Non-Hispanic Black	2739(20.42%)	2672 (22.71%)	67 (4.06%)	
Mexican American	2620(19.53%)	1997 (16.97%)	623 (37.78%)	
Other race	2199(16.39%)	1916 (16.28%)	283 (17.16%)	
Education level, N (%)				< 0.001
Less than high school	3662(27.29%)	2939 (24.98%)	723 (43.84%)	
High school	3183(23.73%)	2788 (23.70%)	395 (23.95%)	
More than high school	6570(48.98%)	6039 (51.33%)	531 (32.20%)	
Body Mass Index (kg/m2)	28.13 ± 5.65	27.67 ± 5.35	31.41 ± 6.58	< 0.001
Intake of Energy (kcal/day)	2460.27 ± 992.66	2517.61 ± 1002.29	2051.14 ± 811.07	< 0.001
Intake of Protein (gm/day)	95.79 ± 43.09	97.77 ± 43.63	81.68 ± 35.97	< 0.001
albumin (mg/L)	55.30 ± 450.59	51.85 ± 451.59	79.97 ± 442.66	< 0.001
creatinine (umol/L)	13219.54 ± 7692.18	13483.83 ± 7823.74	11333.74 ± 6369.85	< 0.001
total cholesterol (mmol/L)	5.08 ± 1.08	5.06 ± 1.07	5.22 ± 1.13	< 0.001
HDL-cholesterol (mmol/L)	1.23 ± 0.35	1.24 ± 0.35	1.17 ± 0.32	< 0.001
LDL-cholesterol (mmol/L)	3.08 ± 0.61	3.08 ± 0.61	3.09 ± 0.59	0.168
triglyceride (mmol/L)	1.70 ± 1.14	1.68 ± 1.04	1.89 ± 1.65	< 0.001
Smoking status, N (%)				< 0.001
Yes	7332(53.91%)	6249 (53.11%)	983 (59.61%)	
No	6183(46.09%)	5517 (46.89%)	666 (40.39%)	
Alcohol status, N (%)				< 0.001
Yes	11022(82.16%)	9740 (82.78%)	1282 (77.74%)	
No	2393(17.84%)	2026 (17.22%)	367 (22.26%)	
Hypertension, N (%)				< 0.001
Yes	4727(35.24%)	3856 (32.77%)	871 (52.82%)	
No	8688(64.76%)	7910 (67.23%)	778 (47.18%)	
Diabetes, N (%)				< 0.001
Yes	1647(12.28%)	1221 (10.38%)	426 (25.83%)	
No	11768(87.72%)	10545 (89.62%)	1223 (74.17%)	

Continuous variables were presented as mean ± SD, and the *P* value was derived using a linear regression model. Categorical variables were presented as percentages, and the *P* value was derived through a weighted chi-square test. *WWI* weight-adjusted-waist index, *HDL* high density lipoprotein, *LDL* low density lipoprotein.

**Table 2:** Baseline characteristics of female study participants.

**Characteristics**	Overall	Non-sarcopenia	Sarcopenia	*P*-value
N	13367	11853	1514(11.33%)	< 0.001
20–39 years	5320	5038	282(5.30%)	0.002
40–59 years	5463	4857	606(11.09%)	< 0.001
≥ 60 years	2584	1958	626(24.23%)	< 0.001
WWI (cm/√kg)	11.03 ± 0.85	10.92 ± 0.80	11.87 ± 0.74	< 0.001
Age (years)	45.34 ± 16.34	44.03 ± 15.82	55.56 ± 16.75	< 0.001
Race/ethnicity, N (%)				< 0.001
Non-Hispanic White	5717(42.77%)	5188 (43.77%)	529 (34.94%)	
Non-Hispanic Black	2854(21.35%)	2777 (23.43%)	77 (5.09%)	
Mexican American	2566(19.20%)	1889 (15.94%)	677 (44.72%)	
Other race	2230(16.68%)	1999 (16.86%)	231 (15.26%)	
Education level, N (%)				< 0.001
Less than high school	3256(24.36%)	2554 (21.55%)	702 (46.37%)	
High school	2963(22.17%)	2629 (22.18%)	334 (22.06%)	
More than high school	7148(53.47%)	6670 (56.27%)	478 (31.57%)	
Body Mass Index (kg/m^2^)	29.00 ± 7.17	28.49 ± 7.00	33.00 ± 7.22	< 0.001
Intake of Energy (kcal/day)	1779.63 ± 682.38	1807.34 ± 685.36	1562.62 ± 617.20	< 0.001
Intake of Protein (gm/day)	68.33 ± 29.17	69.14 ± 29.39	62.01 ± 26.63	< 0.001
albumin (mg/L)	37.68 ± 285.7	34.48 ± 249.38	62.71 ± 481.70	< 0.001
creatinine (umol/L)	9949.59 ± 6819.03	10121.46 ± 6911.51	8604.05 ± 5877.07	< 0.001
total cholesterol (mmol/L)	5.12 ± 1.05	5.10 ± 1.04	5.33 ± 1.08	< 0.001
HDL-cholesterol (mmol/L)	1.47 ± 0.40	1.48 ± 0.41	1.40 ± 0.35	< 0.001
LDL-cholesterol (mmol/L)	3.00 ± 0.60	2.99 ± 0.60	3.08 ± 0.62	< 0.001
triglyceride (mmol/L)	1.39 ± 0.80	1.36 ± 0.80	1.56 ± 0.79	< 0.001
Smoking status, N (%)				< 0.001
Yes	4842(36.22%)	4378 (36.94%)	464 (30.65%)	
No	8525(63.78%)	7475 (63.06%)	1050 (69.35%)	
Alcohol status, N (%)				< 0.001
Yes	8320(62.24%)	7582 (63.97%)	738 (48.75%)	
No	5047(37.76%)	4271 (36.03%)	776 (51.25%)	
Hypertension, N (%)				< 0.001
Yes	4741(35.47%)	3920 (33.07%)	821 (54.23%)	
No	8626(64.53%)	7933 (66.93%)	693 (45.77%)	
Diabetes, N (%)				< 0.001
Yes	1640(12.27%)	1273 (10.74%)	367 (24.24%)	
No	11727(87.73%)	10580 (89.26%)	1147 (75.76%)	

Continuous variables were presented as mean ± SD, and the *P* value was derived using a linear regression model. Categorical variables were presented as percentages, and the *P* value was derived through a weighted chi-square test. *WWI* weight-adjusted-waist index, *HDL* high density lipoprotein, *LDL* low density lipoprotein.

### The association between WWI and sarcopenia

Table 3 illustrates a clear positive correlation between WWI and the occurrence of sarcopenia. Our findings strongly establish a positive and statistically significant correlation between WWI, treated as a continuous variable, and the likelihood of sarcopenia across all three models for both males and females. Among males in the fully adjusted model, a one-unit increase in WWI corresponds to a 14.55-fold higher risk of sarcopenia development (OR: 14.55, 95% CI 12.33, 17.15). Furthermore, this correlation retains statistical significance even when WWI is categorized into four quartiles. Individuals in the highest WWI quartile exhibited a significantly elevated risk of sarcopenia in comparison to those in the lowest quartile (OR: 124.79, 95% CI 50.89, 305.99; *P* for trend < 0.0001). Similarly, among females in the fully adjusted model, a one-unit increase in WWI corresponds to a 2.86-fold higher risk of sarcopenia development (OR: 2.86, 95% CI 2.59, 3.15). Individuals in the highest WWI quartile showed a significantly heightened risk of sarcopenia compared to those in the lowest quartile (OR: 9.17, 95% CI 6.54, 12.86; *P* for trend < 0.0001).

**Table 3:** The associations between WWI and sarcopenia.

	Model 1 [OR (95% CI)]	Model 2 [OR (95% CI)]	Model 3 [OR (95% CI)]
Male			
WWI (continuous)	15.50 (13.69, 17.56) < 0.0001	15.75 (13.66, 18.15) < 0.0001	14.55 (12.33, 17.15) < 0.0001
WWI (quartile)			
Q1(8.23–10.21)	Reference	Reference	Reference
Q2(10.21–10.75)	9.92 (3.95, 24.93) < 0.0001	6.53 (2.60, 16.44) < 0.0001	5.19 (2.06, 13.10) 0.0005
Q3(10.75–11.26)	64.08 (26.44, 155.31) < 0.0001	36.34 (14.94, 88.36) < 0.0001	24.96 (10.20, 61.05) < 0.0001
Q4(11.26–13.83)	424.74 (176.19, 1023.93) < 0.0001	225.59 (93.05, 546.91) < 0.0001	124.79 (50.89, 305.99) < 0.0001
*P* for trend	< 0.0001	< 0.0001	< 0.0001
Female			
WWI (continuous)	4.47 (4.13, 4.85) < 0.0001	3.55 (3.25, 3.88) < 0.0001	2.86 (2.59, 3.15) < 0.0001
*WWI (quartile)*			
Q1(7.90–10.42)	Reference	Reference	Reference
Q2(10.42–11.00)	3.78 (2.67, 5.36) < 0.0001	2.81 (1.98, 4.00) < 0.0001	2.28 (1.60, 3.27) < 0.0001
Q3(11.00–11.60)	9.45 (6.81, 13.11) < 0.0001	5.76 (4.13, 8.04) < 0.0001	3.86 (2.75, 5.43) < 0.0001
Q4(11.60–15.52)	33.02 (24.05, 45.33) < 0.0001	16.51 (11.93, 22.86) < 0.0001	9.17 (6.54, 12.86) < 0.0001
*P* for trend	< 0.0001	< 0.0001	< 0.0001

Model 1: no covariates were adjusted. Model 2: age, race, and education level were adjusted. Model 3: age, race, education level, body mass index, intake of energy, intake of protein, albumin, creatinine, total cholesterol, HDL-cholesterol, LDL-cholesterol, triglyceride, smoking status, alcohol status, hypertension, and diabetes were adjusted. *OR* odds ratio, 95% CI 95% confidence interval, *WWI* weight-adjusted-waist index.

### Subgroup analysis

We explored the association between WWI and sarcopenia across diverse demographic groups through subgroup analyses and interaction tests focusing on gender, age, BMI, hypertension, and diabetes. As shown in Table 4, the likelihood of sarcopenia development notably escalates with elevated WWI among various male groups. It is noteworthy that distinctions exist among subgroups stratified by BMI. In male populations categorized as Obesity Class III, for each incremental unit in WWI, the risk of sarcopenia development merely increases by a factor of 3.87 compared to previous levels. In the female population, except for underweight females, there is a positive correlation between WWI and the risk of developing sarcopenia in all other groups. When grouped by age and the presence of hypertension, although a positive correlation exists between WWI and the risk of developing sarcopenia, statistically significant differences between groups also emerge. In the age groups of 20–39 and 40–59, a one-unit increase in WWI corresponded to a 3.52 and 3.42 times higher risk of developing sarcopenia, respectively. Conversely, in individuals aged 60 and above, each one-unit increase in WWI correlated with a 2.10 times higher risk of developing sarcopenia. For individuals with hypertension, each one-unit increase in WWI was associated with a 2.30 times higher risk of sarcopenia, while among those without hypertension, each one-unit increase in WWI corresponded to a 3.60 times higher risk of sarcopenia.

**Table 4:** Subgroup analysis of the association between WWI and sarcopenia.

Subgroup	OR (95% CI)	*P* value	*P* for interaction
Male			
Age			0.2088
20–39 years	13.77 (10.47, 18.11)	< 0.0001	
40–59 years	17.32 (13.43, 22.32)	< 0.0001	
≥ 60 years	13.01 (10.13, 16.72)	< 0.0001	
BMI			< 0.0001
Underweight	12.09 (1.91, 76.49)	0.0081	
Normal weight	12.95 (8.75, 19.16)	< 0.0001	
Overweight	17.80 (13.90, 22.80)	< 0.0001	
Obesity class I	17.98 (12.69, 25.49)	< 0.0001	
Obesity class II	16.08 (9.69, 26.69)	< 0.0001	
Obesity class III	3.87 (2.37, 6.30)	< 0.0001	
Hypertension			0.4360
Yes	15.44 (12.34, 19.32)	< 0.0001	
No	13.85 (11.28, 17.00)	< 0.0001	
Diabetes			0.9884
Yes	14.57 (10.56, 20.12)	< 0.0001	
No	14.54 (12.17, 17.37)	< 0.0001	
Female			
Age			< 0.0001
20–39 years	3.52 (2.89, 4.29)	< 0.0001	
40–59 years	3.42 (2.93, 3.97)	< 0.0001	
≥ 60 years	2.10 (1.81, 2.43)	< 0.0001	
BMI			0.5438
Underweight	6.97 (0.74, 65.74)	0,0901	
Normal weight	3.29 (2.60, 4.15)	< 0.0001	
Overweight	2.71 (2.27, 3.22)	< 0.0001	
Obesity class I	3.03 (2.50, 3.67)	< 0.0001	
Obesity class II	2.96 (2.32, 3.78)	< 0.0001	
Obesity class III	2.50 (1.95, 3.20)	< 0.0001	
Hypertension			< 0.0001
Yes	2.30 (2.02, 2.62)	< 0.0001	
No	3.60 (3.13, 4.13)	< 0.0001	
Diabetes			0.2629
Yes	2.56 (2.07, 3.17)	< 0.0001	
No	2.93 (2.63, 3.27)	< 0.0001	

age, race, education level, body mass index, intake of energy, intake of protein, albumin, creatinine, total cholesterol, HDL-cholesterol, LDL-cholesterol, triglyceride, smoking status, alcohol status, hypertension, and diabetes were adjusted. *OR* odds ratio, 95% CI 95% confidence interval, *BMI* body mass index.

### The predictive capacity of WWI for sarcopenia

**Figure 2:**
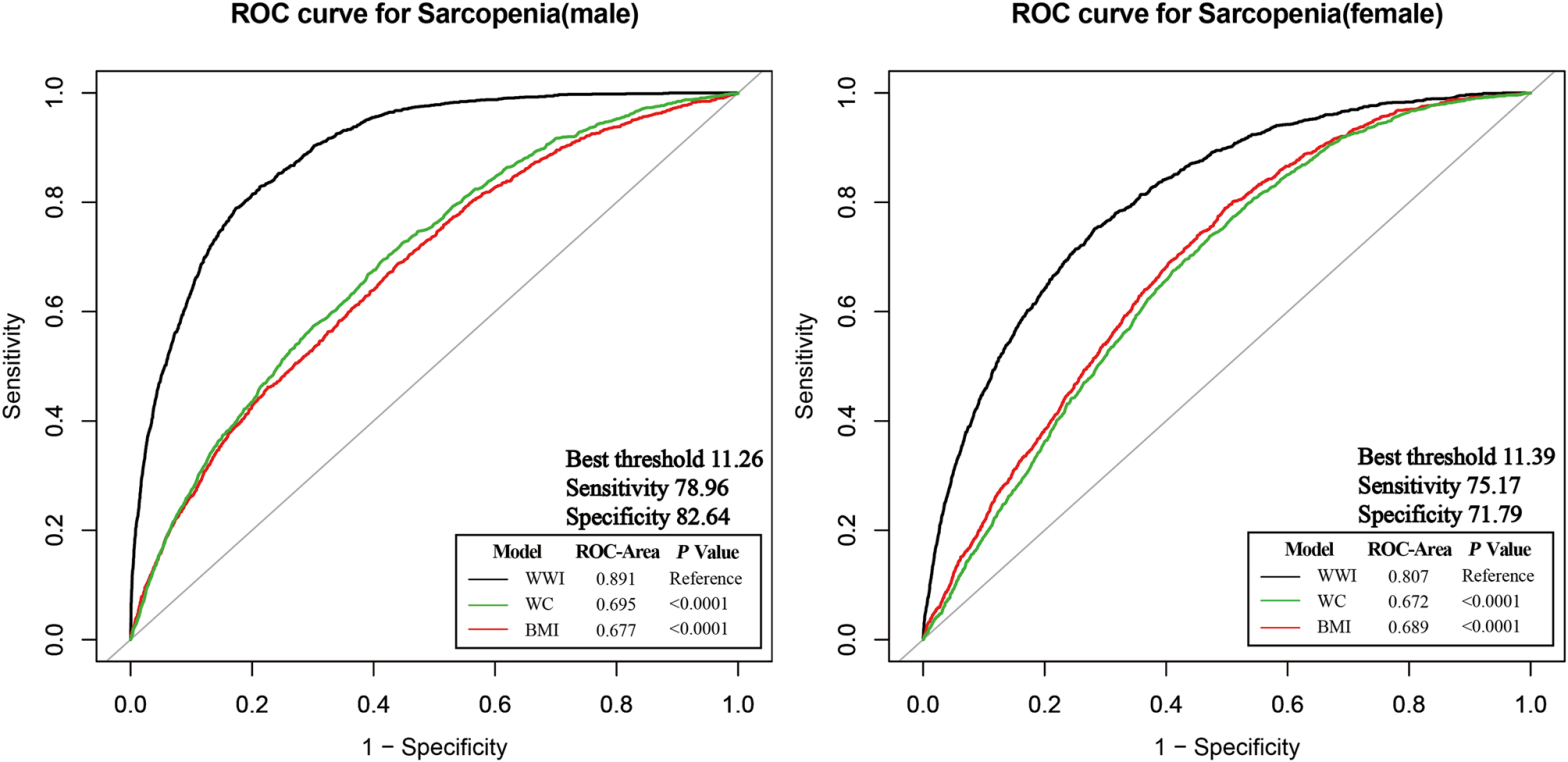
WWI for predicting sarcopenia in male and female. *ROC* receiver operating characteristic, *WWI* weight-adjusted-waist index, *WC* waist circumference, *BMI* body mass index.

Figure 2 displays the ROC curves illustrating the predictive capability of WWI for sarcopenia. The AUC was 0.8910 (95% CI 0.8837–0.8983) for males and 0.8070 (95% CI 0.7961–0.8180) for females. The optimal cutoff value for diagnosing sarcopenia using WWI was 11.26 cm/√kg (sensitivity: 78.96%, specificity: 82.64%) in males and 11.39 cm/√kg (sensitivity: 75.17%, specificity: 71.79%) in females. WWI demonstrated excellent predictive ability for sarcopenia, and was better than other obesity indicators (WC, BMI).

## Discussion

The primary objective of this investigation was to assess the prevalence of sarcopenia among adults and explore its relationship with WWI. In our cross-sectional analysis encompassing a sample of 13,415 male and 13,367 female, our findings indicate that sarcopenia manifests not only in the elderly demographic but also among younger and middle-aged individuals. Moreover, we observed a slightly higher incidence of sarcopenia in males compared to females. Within the male adult cohort, the prevalence of sarcopenia stood at 12.29% (6.17% in the 20–39 age group, 10.32% in the 40–59 age group, 30.11% in the ≥ 60 age group). Correspondingly, among female adults, sarcopenia occurred at a rate of 11.33% (5.30% in the 20–39 age group, 11.09% in the 40–59 age group, 24.23% in the ≥ 60 age group). Additionally, a positive correlation between WWI and sarcopenia was identified in both male and female adults. This positive correlation persists across different subgroups, except for underweight females. Notably, the optimal cut-off value for detecting sarcopenia was 11.26 cm/√kg in male and 11.39 cm/√kg in female.

To the best of our knowledge, this is the first article assessing the correlation between WWI and sarcopenia. Prior research has shown a negative correlation between WWI and both appendicular lean mass and abdominal muscle mass among middle-aged and elderly populations^14–16^. Moreover, among specific disease populations, WWI independently associated with sarcopenic obesity^11,12^. These studies predominantly concentrated on the correlation between WWI and muscle in middle-aged and elderly individuals. In this study, elevated WWI poses a risk factor for sarcopenia in individuals aged over 20 years. By conducting subgroup analysis, it further substantiates the correlation between WWI and sarcopenia across various age groups. Sarcopenia is commonly regarded as an age-related condition, often receiving attention only in the elderly population. But with deeper investigations into sarcopenia, it is believed that muscle loss initiates from the early stages of life^3^. The diagnostic criteria for sarcopenia used in this study were derived from a study with an average age of around 75 years old^21^, whereas the age requirement for individuals included in this study is 20 years old and above, thus creating a discrepancy. This study indicated a prevalence of 5–10% for sarcopenia in both young and middle-aged individuals, consistent with similar conclusions drawn in previous research.^25^. Age is highly correlated with sarcopenia. Therefore, in this study, age is utilized both as a covariate and for stratifying participants into subgroups for analysis. Upon analysis, it was found that even after accounting for the influence of age, the association between WWI and sarcopenia remains robust. Furthermore, subgroup analysis reveals that the relationship between WWI and sarcopenia remains robust across different age groups, further validating the association between WWI and sarcopenia. Sarcopenia should be given due attention from a young age for proper prevention and timely treatment.

The relationship between various obesity indices and sarcopenia is a subject of controversy. This study identified high WWI as a significant risk factor for sarcopenia. Past research suggested that high BMI was a protective factor for sarcopenia^26–29^, while high body fat percentage was a risk factor^28,30,31^. Although these indices indicate a higher degree of obesity as they increase in value, the observed results are inconsistent. High BMI, generally considered detrimental to health, paradoxically exhibits a protective effect against sarcopenia. There exists an "obesity paradox" between BMI and sarcopenia. That might be because these studies primarily focus on the elderly population, where age-related changes in body composition occur, such as increased fat tissue and decreased muscle tissue. This implies that weight and BMI might remain relatively stable while the body fat percentage increases^32,33^. As fat mass increases and muscle mass declines, a phenomenon of fat redistribution takes place. This manifests as fat transfer from subcutaneous regions to the abdominal cavity (visceral fat) and its infiltration into muscles^34–36^. Elevated visceral fat can heighten the risk of sarcopenia^37^. A reciprocal influence exists between the accumulation of visceral fat and the loss of skeletal muscle mass^38^. Sarcopenia diminishes physical activity, lowers energy expenditure, and heightens the risk of obesity^39^. Conversely, increased visceral fat induces inflammation, contributing to sarcopenia development^40^. Due to BMI is inability to differentiate between fat mass and fat-free mass, which possess differing effects on morbidity and mortality risk, using BMI might have limitations. When assessing sarcopenia, priority should be given to evaluating lean mass and fat mass rather than focusing solely on overall body weight. WC is directly linked to visceral fat^41^ and is a simple indicator for assessing visceral fat but doesn’t consider individual weight. WWI is derived from the combined calculation of WC and weight, demonstrating superior predictive capabilities compared to WC and BMI in this study.

Presently, commonly utilized tools for sarcopenia screening encompass questionnaires, serum biomarkers, and anthropometric indices^42^. Although the SARC-F questionnaire is commonly used, its low sensitivity renders it prone to overlooking suspicious cases^8^. Regarding serum biomarkers, commonly utilized ones include serum creatinine and serum cystatin C. Nonetheless, these biomarkers are vulnerable to influences from concurrent diseases, and there is a lack of consensus regarding their diagnostic cutoff values^43^. Calf circumference stands out as the most commonly used anthropometric index, acknowledged for its simplicity and established predictive capability for sarcopenia in specific disease populations^44,45^ and among the elderly^46^. Similarly, WWI functions as a straightforward index applicable across young, middle-aged, and elderly populations. Unfortunately, this study lacks data on calf circumference, preventing a comparison of the predictive abilities of WWI and calf circumference for sarcopenia.

The study exhibits several strengths. Firstly, it relied on NHANES data, ensuring the objectivity of the information. Secondly, we meticulously adjusted for confounding covariates, thereby bolstering the reliability of our findings and their applicability to a wider range of individuals. However, the study also harbors specific limitations. Initially, we couldn't wholly establish the relationship between WWI and sarcopenia due to the study is cross-sectional nature. Hence, further prospective studies with larger sample sizes are crucial to elucidate causality. Additionally, despite adjusting for numerous potential covariates, the impact of other plausible confounding factors could not be completely eliminated. Finally, concerning the diagnosis of sarcopenia, various countries and regions have their own diagnostic criteria. Given that the data utilized in this study are sourced from a publicly available database in the United States, we opted for the standards set by the National Institutes of Health. Different diagnostic criteria may affect the correlation between WWI and sarcopenia.

## Conclusion

The study suggests that individuals with a higher WWI may have an increased susceptibility to sarcopenia, and a high WWI serves as a risk factor for sarcopenia. This relationship remains consistent across all genders, age groups, and levels of obesity, except for underweight females. Consequently, evaluation of WWI could assist in identifying individuals at risk of sarcopenia. Sarcopenia has a relatively high incidence among young people, and those with higher WWI should focus on exercising to prevent muscle wasting. We have proposed cutoff values of 11.26 cm/√kg for males and 11.39 cm/√kg for females as indicative of sarcopenia. WWI has the potential to serve as a straightforward indicator for self-screening sarcopenia. Individuals can initially utilize WWI for self-assessment and then pursue additional testing using bioelectrical impedance analysis or DEXA as needed.

## Data Availability

The survey data are publicly available on the internet for data users and researchers throughout the world (www.cdc.gov/nchs/nhanes/).
